# Age‐specific reference intervals for routine biochemical parameters in healthy neonates, infants, and young children in Iran

**DOI:** 10.1111/jcmm.17646

**Published:** 2022-12-16

**Authors:** Niloufar Abdollahian, Hamideh Ghazizadeh, Maryam Mohammadi‐Bajgiran, Mehran Pashirzad, Mahdiyeh Yaghooti Khorasani, Mary Kathryn Bohn, Shannon Steele, Fatemeh Roudi, Atieh Kamel Khodabandeh, Sara Ghazi Zadeh, Iman Alami‐Arani, Seyede Negin Badakhshan, Habibollah Esmaily, Gordon A. Ferns, Reza Assaran‐Darban, Khosrow Adeli, Majid Ghayour‐Mobarhan

**Affiliations:** ^1^ Department of Biology Mashhad Branch, Islamic Azad University Mashhad Iran; ^2^ CALIPER Program, Division of Clinical Biochemistry, Pediatric Laboratory Medicine The Hospital for Sick Children Toronto Ontario Canada; ^3^ Department of Laboratory Medicine & Pathobiology University of Toronto Toronto Ontario Canada; ^4^ International UNESCO Center for Health‐Related Basic Sciences and Human Nutrition Mashhad University of Medical Sciences Mashhad Iran; ^5^ Social Determinants of Health Research Center Mashhad University of Medical Sciences Mashhad Iran; ^6^ Brighton & Sussex Medical School Division of Medical Education Sussex UK

**Keywords:** biochemical markers, pediatric, reference intervals

## Abstract

Age and sex need to be considered in the establishment of reference intervals (RIs), especially in early life when there are dynamic physiological changes. Since data for important biomarkers in healthy neonates and infants are limited, particularly in Iranian populations, we have determined age‐specific RIs for 7 laboratory biochemical parameters. This cross‐sectional study comprised a total of 344 paediatric participants (males: 158, females: 186) between the ages of 3 days and 30 months (mean age: 12.91 ± 7.15 months). Serum levels of creatinine, urea, uric acid, calcium, phosphate, vitamin D and high‐sensitivity C‐reactive protein (hs‐CRP) were measured using an Alpha classic‐AT plus auto‐analyser. We determined age‐specific RIs using CLSI Ep28‐A3 and C28‐A3 guidelines. No sex partitioning was required for any of the biomarkers. Age partitioning was required for kidney function tests and phosphate. The serum concentration of urea and creatinine increased with age, while phosphate and uric acid decreased with age. Age partitioning was not required for serum calcium, vitamin D, and hs‐CRP, which remained relatively constant throughout the age range. Age‐specific RIs for 7 routine biochemical markers were determined to address critical gaps in RIs in early life to help improve clinical interpretation of blood test results in young children, including neonates. Established age partitions demonstrate the biochemical changes that take place during child growth and development. These novel data will ultimately better disease management in the Iranian paediatric population and can be of value to clinical and hospital laboratories with similar populations.

## 1 INTRODUCTION

Clinical decision‐making relies on the availability of defined reference intervals (RIs) for the appropriate interpretation of laboratory markers of health and disease. According to the International Federation of Clinical Chemistry and Laboratory Medicine (IFCC) guidelines, due to critical gaps (e.g., sex, age, ethnicity), it is essential to determine RIs for biochemical tests and consider age, and sex,^1^ especially in early life. Medical assessment during this period is essential for the timely identification of acute and chronic pathophysiology that may adversely impact growth and development. Dynamic changes in biological, physiological, and hormonal factors are expected to markedly influence biochemical RIs in early infancy.^2^ As a result, while accurate and robust age‐specific RIs for key metabolic, renal, hepatic, macro, and mineral laboratory tests have been determined across most of the paediatric age range,^3^ data in neonates and infants is lacking. Indeed, recent RI studies from national initiatives around the world have emphasized the importance of addressing gaps in RIs for special age and/or sex groups to improve clinical decision‐making in the prognosis and diagnosis of disease. In this study, we have determined age‐specific RIs for 7 routine laboratory biochemical parameters in healthy Iranian neonates and infants.

## 2 METHODS

In this cross‐sectional study, 249 females and 231 males aged 3 days–30 months were enrolled upon informed consent. After applying the inclusion and exclusion criteria,^4^ 344 paediatric participants were included (males: 158, females: 186 and mean age: 12.91 ± 7.15 months) Creatinine, urea, uric acid, calcium, phosphate, vitamin D (25 (OH)), and hs‐CRP were measured using commercially available kits (Pars Azmoun) on an Alpha classic‐AT plus auto‐analyser (TS‐Technology, Tajhizat Sanjesh Co., Ltd.). The intra‐ and inter‐assay coefficients of variation (CV) and mean were 0.4% and 31.64 mg/dl for urea, 0.2% and 3.27 mg/dl for phosphate, 2% and 4.55 mg/dl for uric acid, 5% and 1.31 mg/dl for creatinine, 0.1% and 9.96 for calcium, and 10.0% and 6 mg/dl for hs‐CRP, respectively. We determined age‐specific RIs using CLSI Ep28‐A3 guidelines. Selected partitions were evaluated statistically by the Harris and Boyd method.^5^ Outliers were removed using the Tukey or Adjusted Tukey method, depending on the distribution. To obtain RIs, defined as the 2.5th and 97.5th percentiles, we used a nonparametric rank method or the robust statistical algorithm, depending on sample size. The lower and upper reference limits were considered and corresponding 90% confidence intervals were calculated.^6^, ^7^

## 3 RESULTS

**FIGURE 1:**
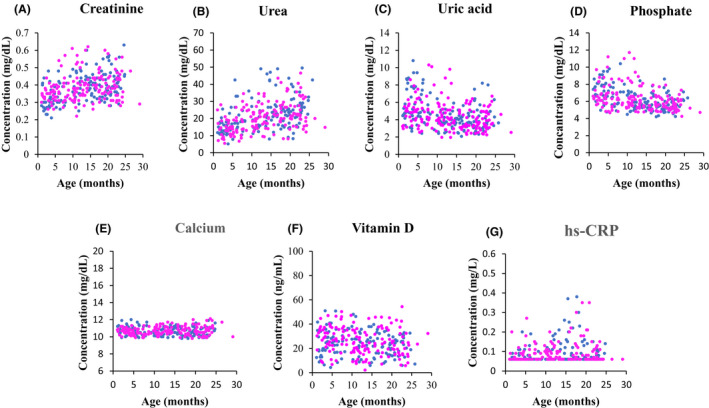
Scatterplot distributions for creatinine (A), urea (B), and uric acid (C), phosphate (D), calcium (E), vitamin D (F) and hs‐CRP (G) for children aged 3 days to 30 months. Females and males are shown by pink and blue circles, respectively.

Age specific distributions for all evaluated biochemical tests across the age range (3 days–30 months) are provided in Figure 1 with outliers removed. Age‐specific RIs for 7 biochemical analytes were calculated and are presented in Table 1. Of the studied biochemical analytes, four demonstrated statistically significant age‐specific differences and none demonstrated sex‐specific differences.

**TABLE 1:** Age‐specific reference intervals for serum biochemical tests

Analyte	Age (months)	Sample size	Male and female reference interval			
			Lower limit	Upper limit	Lower limit confidence interval 90%	Upper limit confidence interval 90%
Creatinine (mg/dl)	0–<5	57	0.22	0.44	0.20–0.24	0.42–0.46
	5–<30	248	0.27	0.57	0.26–0.28	0.55–0.63
Urea (mg/dl)	0–<5	59	6.20	25.62	5.11–7.37	23.15–27.78
	5–<10	48	6.94	38.12	5.71–8.36	34.08–42.71
	10–<30	204	9.03	46.43	6.77–9.96	43.87–50.43
Uric acid (mg/dl)	0–<5	58	2.66	10.17	2.41–2.95	9.32–11.16
	5–<30	257	2.30	8.20	2.10–2.40	7.50–9.80
Calcium (mg/dl)	0–<30	290	9.90	11.84	9.90–9.90	11.79–12.09
Phosphate (mg/dl)	0–<5	60	4.87	10.43	4.62–5.15	9.72–11.12
	5–<10	44	4.48	10.27	4.23–4.73	9.50–11.24
	10–<15	72	4.60	9.75	4.45–4.74	8.94–10.63
	15–<30	129	4.26	7.87	3.86–4.31	7.32–8.45
Vitamin D (ng/ml)	0–<30	289	6.30	46.40	5.02–6.97	43.60–48.47
hsCRP (mg/L)	0–<30	298	0.06	0.26	–	0.18–0.31

Urea and phosphate required age partitioning at 5, 10, and 15 months of age. Urea demonstrated statistically significant increases in concentration with age. In contrast, phosphate concentrations decreased with age and demonstrated variability in reference values as reflected by wider confidence intervals. Creatinine and uric acid required partitioning only at 5 months of age with lower creatinine and higher uric acid levels reported in infants 0–<5 months relative to those 5–<30 months of age (Table 1). Serum concentrations of calcium, vitamin D, and hs‐CRP remained relatively constant within the first 30 months of life, requiring no age partitions (Figure 1). No statistically significant sex‐specific differences were observed across analytes.

## 4 DISCUSSION

The current study determined age‐ and sex‐specific RIs for several routine biochemical analytes using statistical methodology of CLSI Ep28‐A3 guidelines and CALLIPER. These data report paediatric RIs for biochemical analytes in an Iranian cohort of healthy infants and children aged 3 days–30 months for the first time. Study findings were similar to other reports in different populations, with few exceptions, as described below.

The established RI for creatinine in our study compared most closely to CALLIPER findings in infants aged 15 days to 1 year (0.31–0.53 mg/dl) on the Abbott Architect system^4^ and 0**–**2 years (0.17–0.52 mg/dl) on the Siemens Atellica system.^8^ In contrast to our results, RIs for urea (5.00–20.00 mg/dl) and uric acid (1.60–6.30 mg/dl) in individuals younger than 1 year reported in other studies were lower than our results.^4^, ^8^ These observed discrepancies may be associated with differences in general population characteristics and analytical factors.

In previous Canadian^8^ and Korean studies,^9^ the RI for calcium in healthy participants <2 years old, and 1 year old, respectively, were similar but slightly lower to our results observed in the 5**–**30 months old group. Interestingly, CALLIPER studies showed comparable results with ours.^8^ We also observed in our study and demonstrated variability in RIs as reflected by wider confidence intervals. While statistically significant age‐specific differences were observed for phosphate, clinical significance is unclear particularly given biological variation reported in the literature.^10^ Also, RIs established in mentioned studies had narrower range than those established in the current study. Such discrepancies may be explained by various population characteristics as wel1 as diverse analytical factors.

In comparison to our study, established RIs for vitamin D in young children were generally lower than other studies.^11^ Reasons for differences observed between reported results may be related to different analytical methods and lifestyle (i.e. poor diet and sun exposure). There is a high prevalence of vitamin D deficiency in Asian countries such as Iran,^12^, ^13^, ^14^ with about 30% of Iranian infants having a serum vitamin D level<20 ng/ml.^13^ While the main reason for insufficient serum vitamin D in infants is insufficient exposure to sunlight and breastfeeding without supplementation,^15^ neither of these factors were assessed in the current study.

Established RI for hs‐CRP was lower in our study compared to others.^4^ For example, CALLIPER has reported the need for age partitioning for hs‐CRP interpretation in individuals aged 0–<19 years. Specifically, they demonstrated a substantial age‐related decrease in derived RIs for hs‐CRP, with the upper limits changing from 6.1 mg/L (0–14 days) to 1 mg/L (15 days–<15 years) with age. These estimates are higher than the upper limit of 0.21 mg/L derived in our study. In line with our results, sex partitioning was not required. Differential results observed between these studies are likely due to different analytical methods and particularly the lack of very young infants in our cohort (<1 month). Population factors such as body mass index of participants may also play a role.

### 4.1 Limitations

This study has selected limitations. First, the sample size for establishing RIs was limited, particularly in the first month of life where immense physiological changes occur as part of development. This should be considered when interpreting findings and applying them to postnatal populations. It is generally recommended that at least 120 individuals are included in RI calculations in order to calculate the 90% CIs using the recommended nonparametric approach. In our study, sample sizes fell below this recommendation only in select age partitions (0–<5, 5–<10, and 10–<15 years). In addition, some differences reported between our findings and other studies might be explained by specific analytical platforms and commercial kits applied in our study. All RIs should be validated for use in clinical laboratories using different analytical platforms and local populations prior to implementation as per CLSI EP28‐A3c guidelines.

Taken together, these novel data will ultimately assist clinical laboratory test interpret in the Iranian paediatric population and can be of value to clinical and hospital laboratories with similar populations.

## AUTHOR CONTRIBUTIONS

**Niloufar Abdollahian:** Data curation (equal); investigation (equal); writing – original draft (equal). **Hamideh Ghazizadeh:** Conceptualization (equal); formal analysis (equal); methodology (equal); software (equal); writing – original draft (equal). **Maryam Mohammadi ajgiran:** Data curation (equal); investigation (equal). **Mehran Pashirzad:** Data curation (equal); writing – original draft (equal). **Mahdiyeh Yaghooti Khorasani:** Data curation (equal); writing – original draft (equal). **Mary Kathryn Bohn:** Methodology (equal); writing – original draft (equal); writing – review and editing (equal). **Shannon Steele:** Writing – review and editing (equal). **Fatemeh Roudi:** Validation (equal); visualization (equal). **Atieh Kamel Khodabandeh:** Data curation (equal); formal analysis (equal); investigation (equal); methodology (equal). **Sara Ghazizadeh:** Data curation (equal). **Iman Alami Arani:** Data curation (equal). **Seyede Negin Badakhshan:** Investigation (equal). **Habibollah Esmaeili:** Resources (equal); software (equal); validation (equal); visualization (equal). **Gordon A Ferns:** Writing – review and editing (equal). **Reza Assaran Darban:** Data curation (equal); investigation (equal); visualization (equal). **Khosrow Adeli:** Investigation (equal); methodology (equal); project administration (equal); software (equal); supervision (equal); validation (equal); visualization (equal); writing – review and editing (equal). **Majid Ghayour Mobarhan:** Funding acquisition (equal); project administration (equal); resources (equal); supervision (equal).

## CONFLICT OF INTEREST

The authors have no conflict of interest to disclose.

## Data Availability

The data that support the findings of this study are available from the corresponding author upon reasonable request.
